# Reconstruction of Chest Wall by Cryopreserved Sternum Allograft After Resection of Sternal Hemangioma: A Case Report

**DOI:** 10.3389/fsurg.2022.796806

**Published:** 2022-03-02

**Authors:** Farahnaz Sadegh Beigee, Ali Sheikhy, Kambiz Sheikhy

**Affiliations:** ^1^Department of Thoracic Surgery, Lung Transplantation Research Center, National Research Institute of Tuberculosis and Lung Diseases, Masih Daneshvari Hospital, Shahid Beheshti University of Medical Sciences, Tehran, Iran; ^2^Research Department, Tehran Heart Center, Tehran University of Medical Sciences, Tehran, Iran; ^3^Non-communicable Disease Research Center, Endocrinology and Metabolism Population Sciences Institute, Tehran University of Medical Sciences, Tehran, Iran

**Keywords:** sternal tumor, hemangioma, sternal allograft, cryopreserved bone allograft, case report

## Abstract

A 5-year-old girl was referred to our department for a mass of sternum that was previously biopsied and diagnosed as hemangioma. Chest X-ray and CT scan confirmed a large sternal mass. We resected the sternum completely and reconstructed a large anterior chest wall defect by a cryopreserved sternal allograft. In the follow-up of the patient, there was no instability of the chest wall and acceptable cosmetic results.

## Introduction

Radical resection of the sternum is indicated in different situations, such as tumors, infection, radionecrosis, and trauma. In addition, it may be considered as an alternative surgical treatment in chest wall deformity (1). Sternum hemangioma is a rare benign bone tumor, and, to the best of our knowledge, about five cases have been reported in the literature, including adult and pediatric; meanwhile, any of them did not include pectus carinatum deformity coexisting with hemangioma of the sternum (2–5). In the case of sternal resection, reconstruction of the sternum is mandatory to prevent flail chest and ventilation impairment; besides, cosmetic consequences must also be considered. Due to mentioned difficulties, this procedure is challenging for most thoracic and reconstructive surgeons (6, 7).

Various techniques have been described previously, but there is no gold standard procedure for managing these defects (8). There is limited experience with a new method for the reconstruction of the sternum by utilizing cryopreserved sternal allograft; furthermore, no previous study was conducted in the pediatric group.

In this case report, we described a 5-year-old girl with the sternum hemangioma and pectus carinatum deformity, on whom reconstruction of the chest wall with a cryopreserved sternal allograft was conducted after complete surgical resection of the tumor (sternectomy).

## Case Report

**Figure 1:**
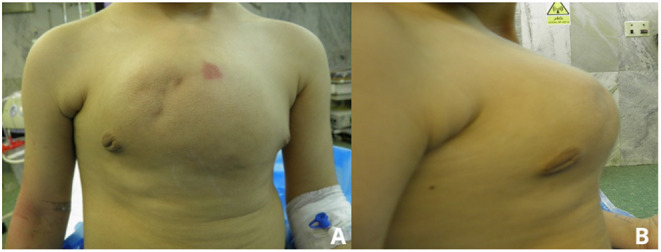
Preoperative anterior **(A)** and lateral **(B)** view pictures showing a deformity and large mass.

**Figure 2:**
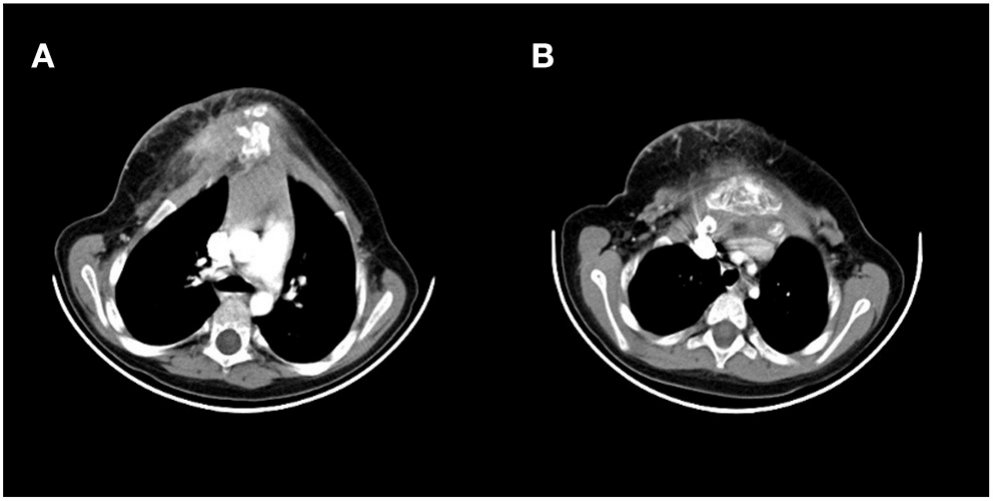
Preoperative CT scan showing a large sternum mass. (**A)** Uppers segments, **(B)** lower segments.

A 5-year-old girl was referred to our thoracic surgery unit following diagnosis of hemangioma of the sternum (Figure 1). When she was 3 years old, following an attack of seizure, her brain MRI revealed a calcified hemangioma in addition to pectus carinatum deformity and thrombocytopenia (platelet count = 15,000/μL) in her primary evaluations. A chest CT scan was performed, which demonstrated a sternal expansile mass (Figure 2). Incisional biopsy of the mass was performed, and pathologic finding was compatible with hemangioma. Considering her thrombocytopenia, she underwent corticosteroid therapy. She experienced recurrent thrombocytopenia after corticosteroids withdrawal; therefore, she was referred for resection of sternal hemangioma as a potential genesis of thrombocytopenia. At the time of admission, she had a platelet count of 20,000/μL eventually; corticosteroid therapy was established before resection to normalize the platelet count.

**Figure 3:**
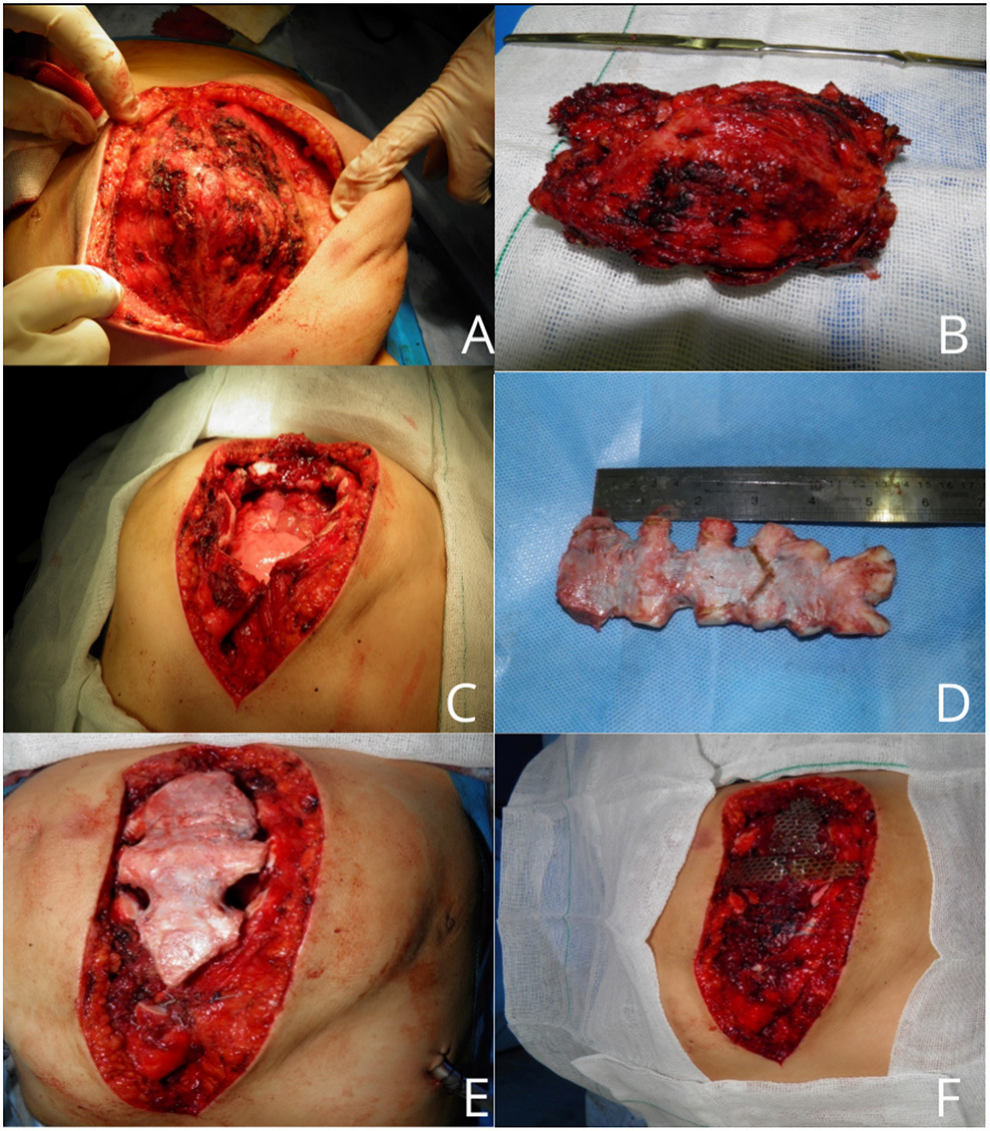
An intraoperative picture of sternal mass **(A)**. Resected hemangioma with an acceptable margin **(B)**. Large chest wall defect **(C)**. Cryopreserved sternal allograft **(D)**. Covering of chest wall defect with graft **(E,F)**.

**Figure 4:**
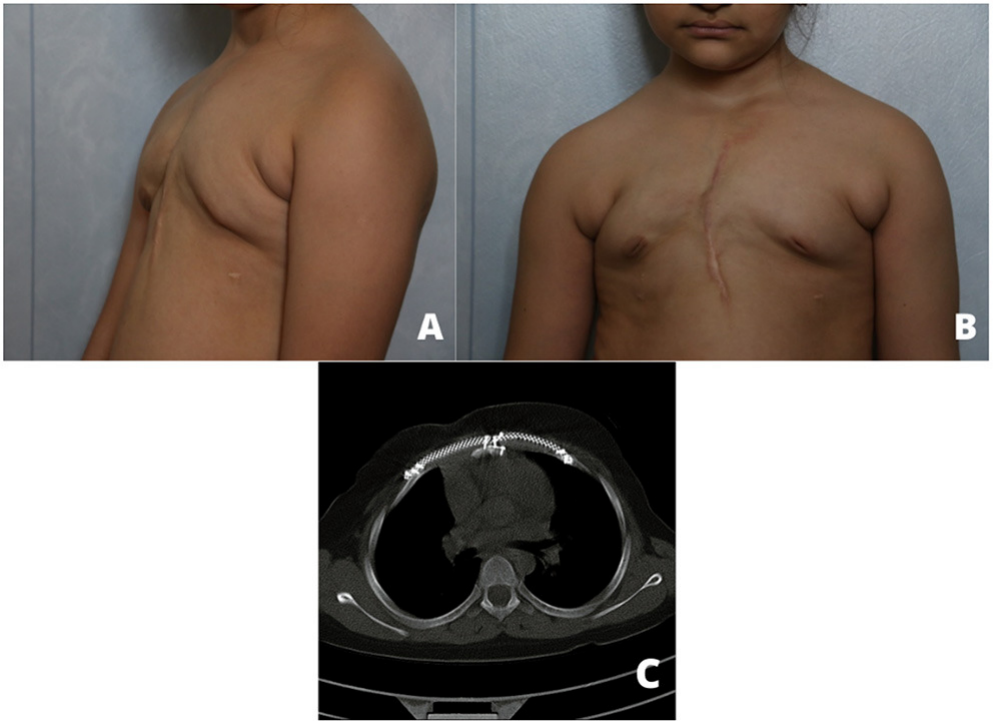
An anterior, lateral photograph **(A,B)** and CT scan **(C)** 16 months after the operation.

According to CT findings, near-complete resection of the sternum was arranged. Based on predicted sternum size, measured according to anthropometric parameters and the resected area of the chest wall, a cryopreserved sternum was ordered from the tissue bank center. At the time of the surgery, a longitudinally elliptical midline incision was performed, which included a previous biopsy site. There was no infiltration or adhesion of mass to skin and subcutaneous tissue except the biopsied area, and a well-defined, capsulated but vascular mass was dissected from surrounding tissues. Due to the large dimension of the tumor, pectoralis major muscles were pushed laterally to nearly midclavicular lines (Figures 3A,B). The upper border of the manubrium seemed to be normal and preserved. The thoracic cavity was entered on the left side, and near-total sternectomy, including tumor and most parts of deformed cartilages, was achieved. Eventually, there was a 15 × 12-cm defect in the anterior chest wall. The previously preserved cryopreserved sternum allograft was prepared for the reconstruction (Figures 3C,D). The graft was dampened by 40°C normal salines, tailored gradually, fixed to the patient's ribs by titanium plates and screws, and fixed to remained manubrium (Figures 3E,F). Subsequently, the graft was covered by pectoralis muscles and after insertion of a bilateral chest tube and subcutaneous right side vacuum suction drainage; the skin was closed in the midline. The patient was extubated in the operation room. After surgery, the chest wall was completely stable, and there was no need for ventilator support. Chest tubes were removed on the 3rd day after the operation, and there was no evidence of wound infection. The patient was discharged after 7 days. In the 1st month after surgery, the defect was stable with no complications. The final pathologic finding was compatible with hemangioma (Supplementary Figure 1). Sixteen months after the operation, there was a minimal deformity in the anterior chest wall without any evidence of chest wall instability (Figure 4).

## Discussion

Hemangioma of the sternum is an extremely rare benign sternal tumor. To the best of our knowledge, <5 cases have been reported previously (2–5), and simultaneous occurrence of pectus carinatum and sternum hemangioma is a novel condition, although this type of anterior chest wall deformity may be acquired and secondary to abnormal overgrowth of the sternum (9). On the other hand, resection of sternum and repair strategies of the remained defect after resection based on different pathologies is challenging, especially in the pediatric. The most challenging part of the operation is the best reconstructive strategy selection for the reconstruction of the anterior chest wall. Reconstruction of soft tissue defects with various types of flaps is less challenging. The ongoing growth of a child and adverse effects of growth restriction following resection and reconstruction is the most important challenging points of pediatrics in contrast to adults.

Methyl methacrylate meshes and sandwiches, polytetrafluoroethylene (PTFE), and polypropylene patches are routinely used in thoracic surgery for reconstruction. The disadvantages of these prosthetic materials are the risk of infection, dislodgement, and long-term complications, including changes in thoracic morphology and function in pediatric patients (10). Metallic materials, such as titanium clips, plates, and moldable multi-hole plates, have been used for the stabilization of the chest wall (11, 12) and may have the same complications as other prosthetic materials. Turna et al. (13) described reconstruction of anterior chest wall defect after wide resection of this part with a well-designed patient-specific titanium implant. In addition, a hydroxyapatite tricalcium- phosphate compound (ceramic) that can be shaped has been introduced for reconstruction (11).

Due to complications related to using prosthetic material, such as infection, rigidity, and probable growth restriction in children, bone grafts have gained considerable attention in the reconstruction of the chest wall (14). Ribs can be harvested as autografts from the opposite surgical site or can be used as allografts. In extensive chest wall defects, as in sternum tumor resection, bone autograft harvest may lead to significant morbidity (15). A potentially inexhaustible source of bone for the reconstruction of chest wall defects in the human cadaver. The main advantage of bone grafts is their capability of integration with host tissue and chest wall stability without rejection, infection, and migration. Bone graft also acts as a scaffold for osteoprogenitor cells, which migrate within the graft, consequently, new bone will be formed (15). Some authors reported cryopreserved sternum allograft for a limited number of patients after sternum resection (15–18). Dell'Amore described this method and his experiences in fourteen patients, which is the largest group of patients (19). He has also described more limited experiences of other surgeons with this method in other centers (20). In all of these reports, no allograft complications have been reported.

Sternum allograft fixation by Titanium bridges and screws may result in a thoracic cage deformity in long term in a growing child; therefore, studies with longer follow-ups are mandatory to clarify this hypothesis.

Using an autologous flap in chest wall reconstruction is an option that is a complex but safe procedure; nevertheless, it may lead to inadequate functional and cosmetic results, especially in mid- and long-term follow-up. On the other hand, some surgeons have reported prosthetic material for reconstruction of the chest wall in this group of patients with acceptable results, but mid- and long-term results are not available and prosthetic material may interfere with the normal growth of the child (21); therefore, using bone allografts may be more beneficial for children.

In conclusion, in this case, we have reported three rare events occurring simultaneously. First, the rare occurrence of sternal hemangioma; second, the occurrence of sternum hemangioma with a pectus carinatum deformity (that most probably is acquired), and the last one is the use of a novel substitute for sternum reconstruction. Using cryopreserved sternum allograft for the reconstruction of the anterior chest wall after sternum resection is a simple and reproducible method.

To the best of our knowledge, this is the first report of chest wall reconstruction by utilizing cryopreserved sternum in children. The most important consideration in this reconstruction method is the restrictive effects on the sternum. Another question is why we did not try to repair pectus carinatum deformity completely? If we tried to resect more adjacent ribs and cartilages for cosmetic results, remained defect might have been as big as which could force us to use longer titanium bridges for sternum allograft fixation on the remained ribs. Still, we could repair the patient's carinatum deformity in the future to achieve more cosmetic results. Although, 16 months after the surgery, there was a minimal chest wall deformity in comparison with preoperative images. More studies with a larger sample size and longer follow up are mandatory to determine the long-term outcome of this type of transplantation, especially in children.

## Data Availability Statement

The original contributions presented in the study are included in the article/Supplementary Material, further inquiries can be directed to the corresponding author.

## Ethics Statement

The studies involving human participants were reviewed and approved by Shahid Beheshti University Ethical Board. Written informed consent to participate in this study was provided by the participants' legal guardian/next of kin. Written informed consent was obtained from the individual(s), and minor(s)' legal guardian/next of kin, for the publication of any potentially identifiable images or data included in this article.

## Author Contributions

FS contributed in data gathering and drafting. AS contributed in revision and drafting. KS contributed in data gathering and final revision. All authors contributed to the article and approved the submitted version.

## Conflict of Interest

The authors declare that the research was conducted in the absence of any commercial or financial relationships that could be construed as a potential conflict of interest.

## Publisher's Note

All claims expressed in this article are solely those of the authors and do not necessarily represent those of their affiliated organizations, or those of the publisher, the editors and the reviewers. Any product that may be evaluated in this article, or claim that may be made by its manufacturer, is not guaranteed or endorsed by the publisher.
