# Supplementary material for: Reconstruction of Chest Wall by Cryopreserved Sternum Allograft After Resection of Sternal Hemangioma: A Case Report
Source: Front Surg. 2022 Mar 2;9:796806. doi: 10.3389/fsurg.2022.796806 (PMC8924364; doi:10.3389/fsurg.2022.796806)
Supplement: Supplementary file 1 [file Data_Sheet_1.docx]

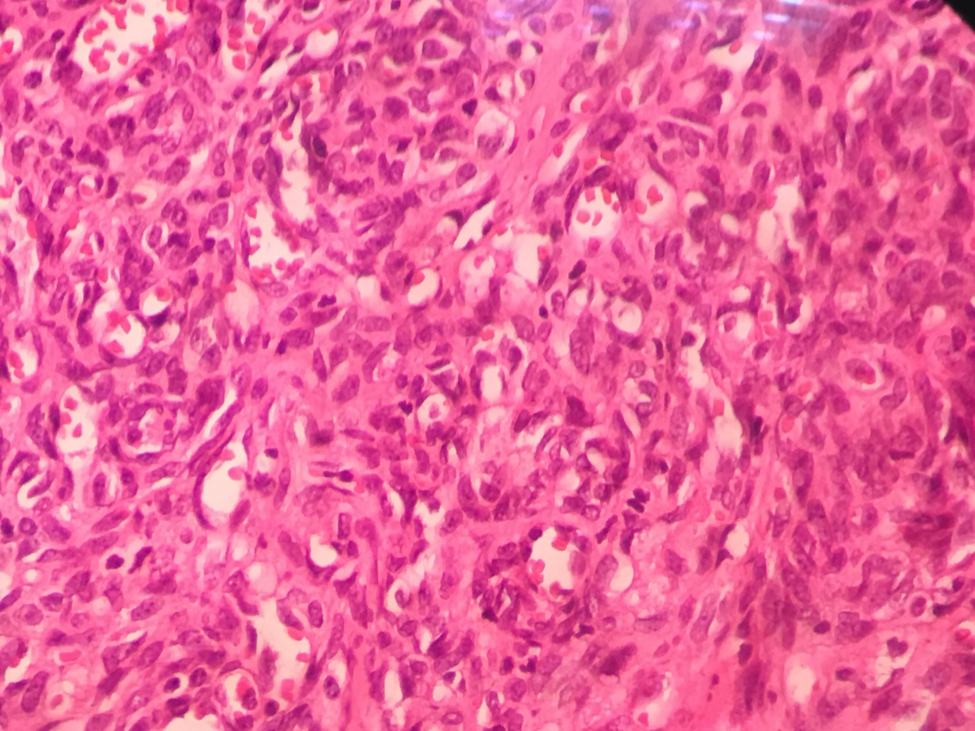


**Figure supp:** **Proliferation of vascular nodules composed of numerous small sized vascular channels containing Red blood cells .**
